# Supplementary material for: Advanced genomics identifies growth effectors for proteotoxic ER stress recovery in Arabidopsis thaliana
Source: Commun Biol. 2022 Jan 11;5:16. doi: 10.1038/s42003-021-02964-8 (PMC8752741; doi:10.1038/s42003-021-02964-8)
Supplement: Supplementary file 2 — Supplementary information [file 42003_2021_2964_MOESM2_ESM.pdf]

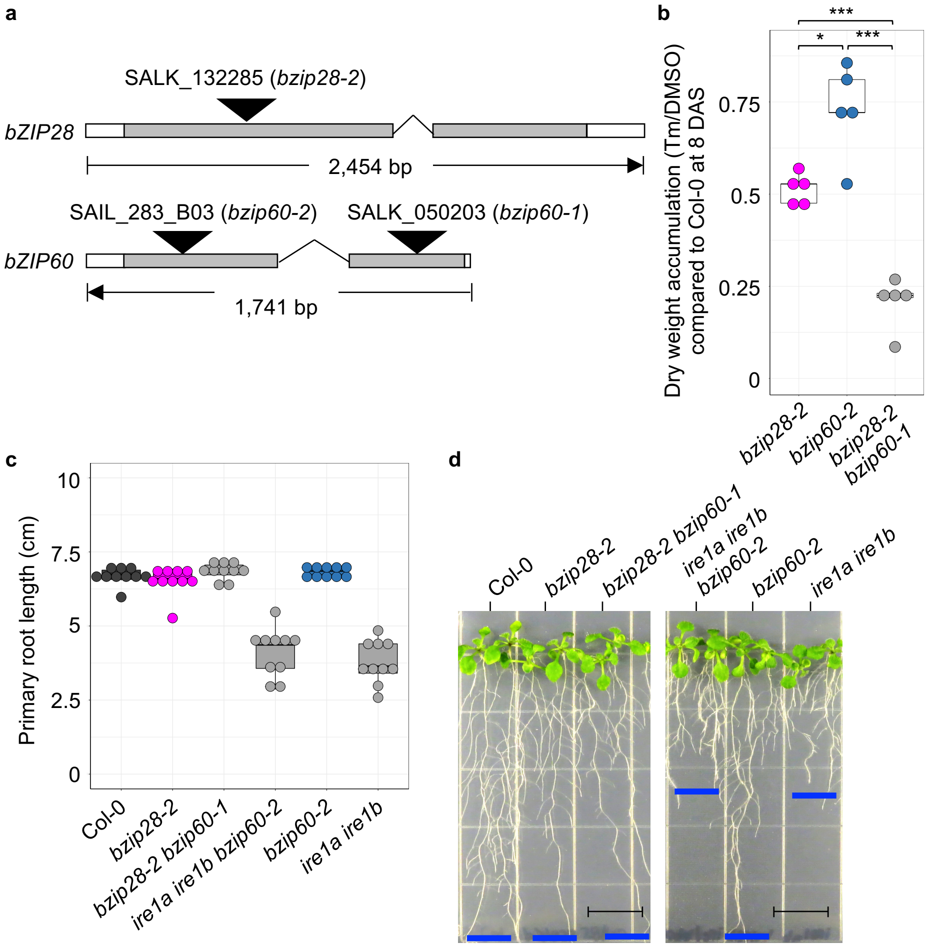

Supplementary Figure 1. Characterization of primary root growth of Col-0, *bzip28-2*, *bzip60-2* and *bzip28-2 bzip60-1* in ER stress recovery. **a** Schematic illustration of insertions of T-DNA in *bZIP28* and *bZIP60*. The allele names are specified with parenthesis. **b** Relative dry weight accumulation in ER stress recovery. The seedlings at 8 DAS in Fig. 1d were collected and analyzed. $n = 5$ (6 seedlings per replicate). $^*P < 0.05$, $^{**}P < 0.01$, $^{***}P < 0.001$ (two-tailed Student's *t*-test). **c**, **d** Comparison of primary root growth among loss-of-function mutants of UPR regulator genes under the normal growth condition. Seeds were sown on growth media plates grown for 12 days. (**c**) Quantification. $n = 10$ (2 seedlings per replicate). (**d**) Representative photos of (**c**). Blue lines indicate the tip of the primary root. Scale bar (black line) = 1.4 cm. In boxplots, each box is bounded by the lower and upper quartiles from which the whisker extends 1.5× the interquartile range, and central bars represent the medians. The source data of **b, c** is provided in Supplementary Data 6.

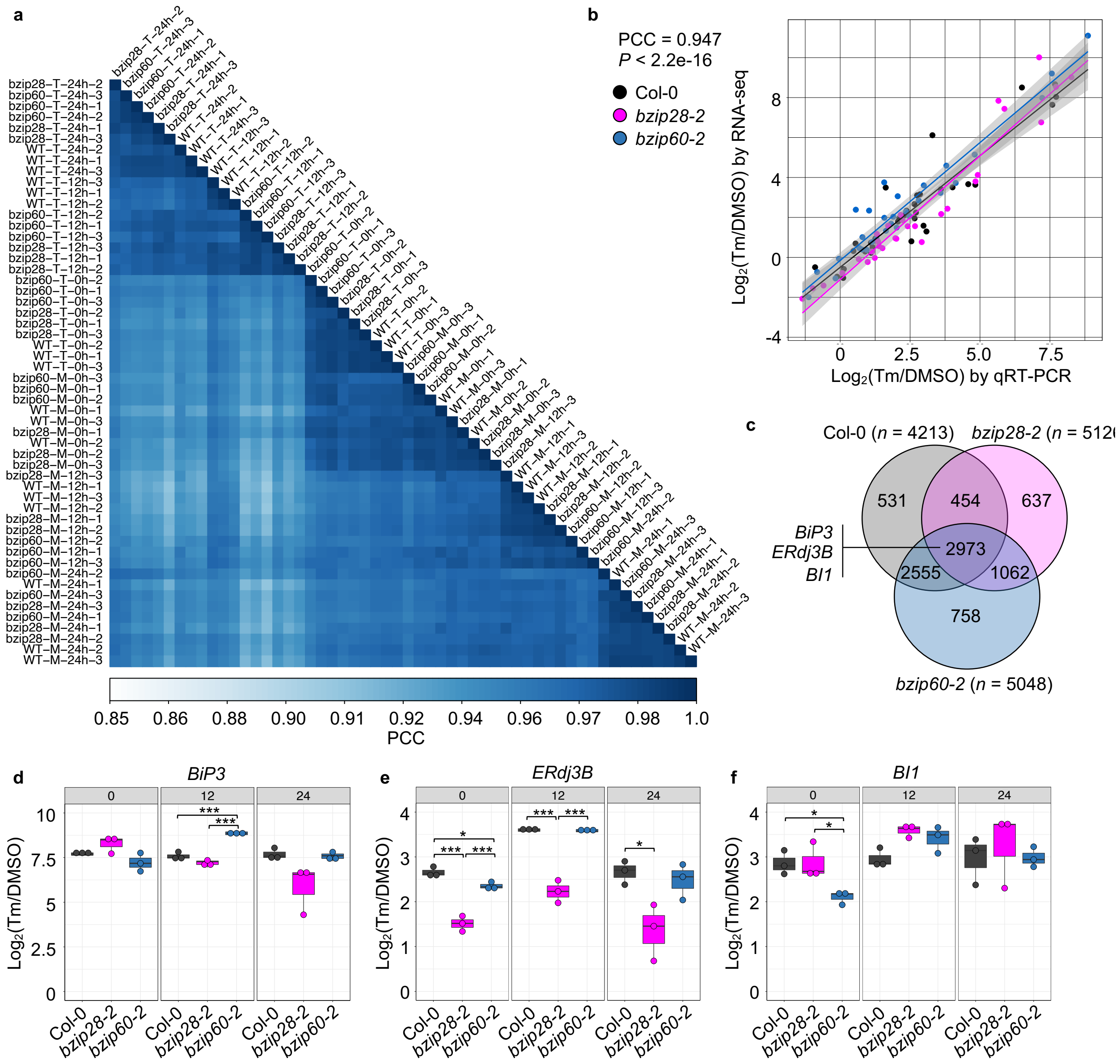

Supplementary Figure 2. High reproducibility of our RNA-seq dataset and Genotype-dependent regulation of UPR biomarker genes during the time-course of ER stress recovery. a Heatmap shows the Pearson correlation coefficient (PCC) of gene expression for all samples analyzed in this study. Genes with fragments per kb exon model per million mapped fragments (FPKM) > 1 in at least one sample were $\log_2$, +1 transformed before the analyses. The color scale indicates the Pearson correlation coefficient. Samples were hierarchically clustered based on their pairwise correlations. T, Tm-treated sample. M, Mock-treated sample. The last number of each sample indicates the order of biological replicate. **b** Validation of the differential expression of the UPR (7 genes) and downstream genes (4 genes). A total of 93 data points of Log_2FC was obtained by qRT-PCR in the same RNA samples used for the RNA-seq. The scatter plot shows a high Pearson correlation coefficient of the values between RNA-seq and qRT-PCR. A full list of genes and primer information is available in Table S1. **c** Venn diagram depicting DEGs in Col-0, *bzip28-2* and *bzip60-2*. The total numbers of DEGs in each genotype are shown in parentheses. **d-f** Expression levels (Log_2[Tm/DMSO]) of *BiP3* (**d**), *ERdj3B* (**e**), *BI1* (**f**) in each genotype at each time-point in the RNA-seq data. $n = 3$ (12 seedlings per replicate). $^*P < 0.05$, $^{***}P < 0.001$ (two-tailed Student's t-test). In boxplots, each box is bounded by the lower and upper quartiles from which the whisker extends 1.5× the interquartile

range, and central bars represent the medians. The source data of **a**, **b** and **d-f** is provided in Supplementary Data 6.

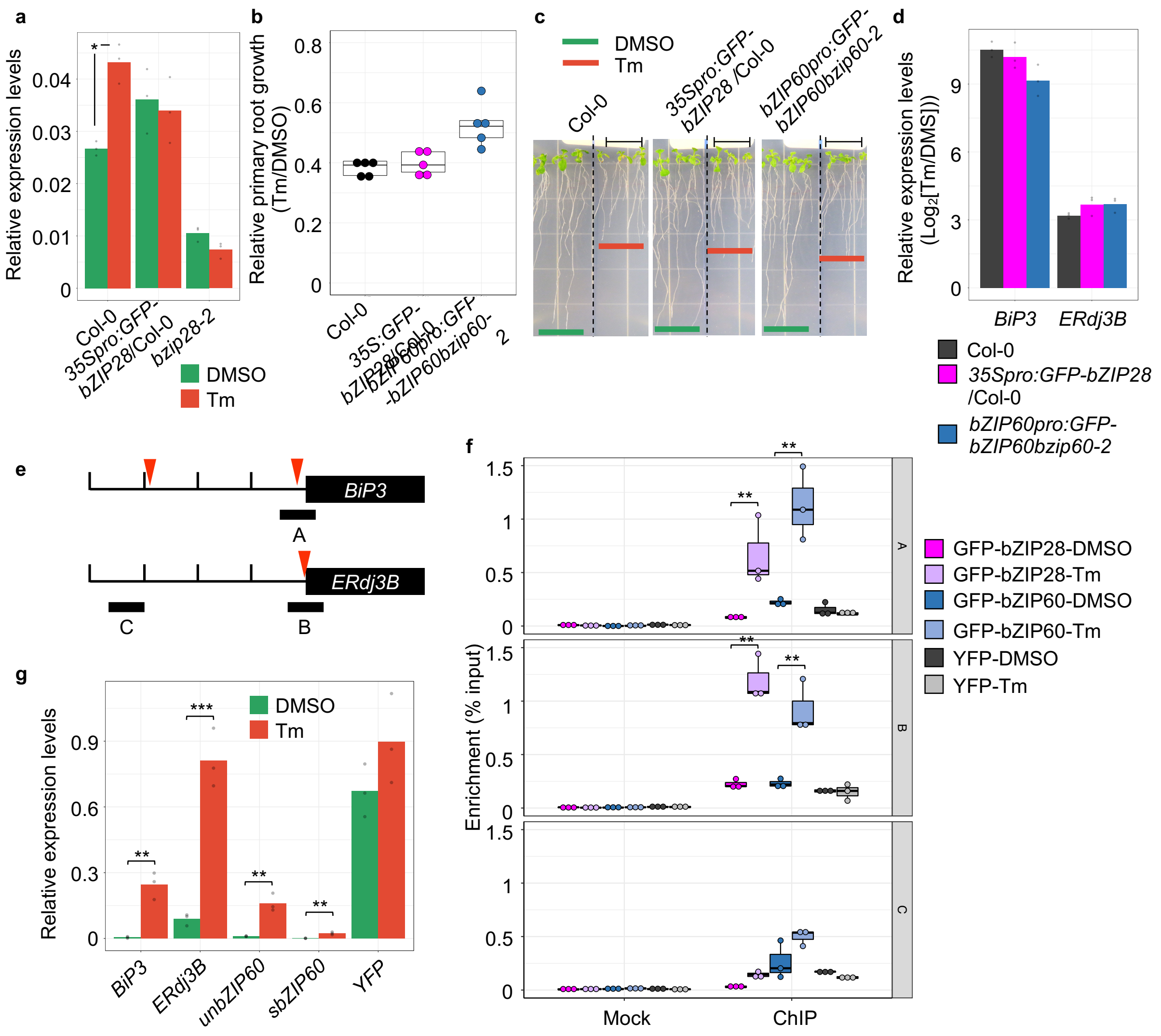

Supplementary Figure 3. Characterization of the GFP-tagged transgenic lines and identification of *in vivo* DNA-binding of bZIP28 and bZIP60 to the promoters of *BiP3* and *ERdj3B* under ER stress using ChIP-qPCR. a qRT-PCR analysis of bZIP28 in Col-0, *35Spro*:*GFP-bZIP28*/Col-0 and *bzip28-2* under ER stress (6 h of Tm or DMSO treatment). *UBQ10* was used as an internal control. *n* = 3 (12 seedlings per replicate). **b, c** Relative root growth of Col-0, *35Spro*:*GFP-bZIP28*/Col-0 and *bZIP60pro*:*GFP-bZIP60*/*bzip60-2* in ER stress recovery. Plants were subjected to ER stress recovery assay described in Fig. 1a. **b** Quantification. *n* = 5 (6 seedlings per replicate). **c** A representative photo. Scale bar (black line), 1.4 cm. **d** qRT-PCR analysis of *BiP3* and *ERdj3B* in Col-0, *35Spro*:*GFP-bZIP28*/Col-0 and *bZIP60pro*:*GFP-bZIP60*/*bzip60-2* under ER stress (6 h of Tm treatment). *n* (number of biological replicate) = 3 (12 seedlings per replicate). **e** A scheme of amplicon locations on the promoters of *BiP3* and *ERdj3B*. The locations of ERSE-I are indicated by red arrowheads. **f** ChIP-qPCR analysis of the binding of bZIP28 and bZIP60 to the promoters of *BiP3* and *ERdj3B* under ER stress (6 h of Tm treatment). *n* (number of biological replicate) = 3 (~100 seedlings per replicate). **g** Expression of UPR genes and YFP in *35Spro*:*YFP*/Col-0 plants under ER stress (6 h of Tm or DMSO treatment), showing the constitutive expression of YFP. *n* = 3 (12 seedlings per replicate). **a, f, g** **P* < 0.05, ***P* < 0.01, ****P* < 0.001, NS = non-significant (two-tailed Student's *t*-test). In boxplots, each box is bounded by the lower and upper quartiles from which the whisker extends 1.5× the interquartile range, and central bars represent the medians. The source data of **a, b, d, f, g** is provided in Supplementary Data 6.

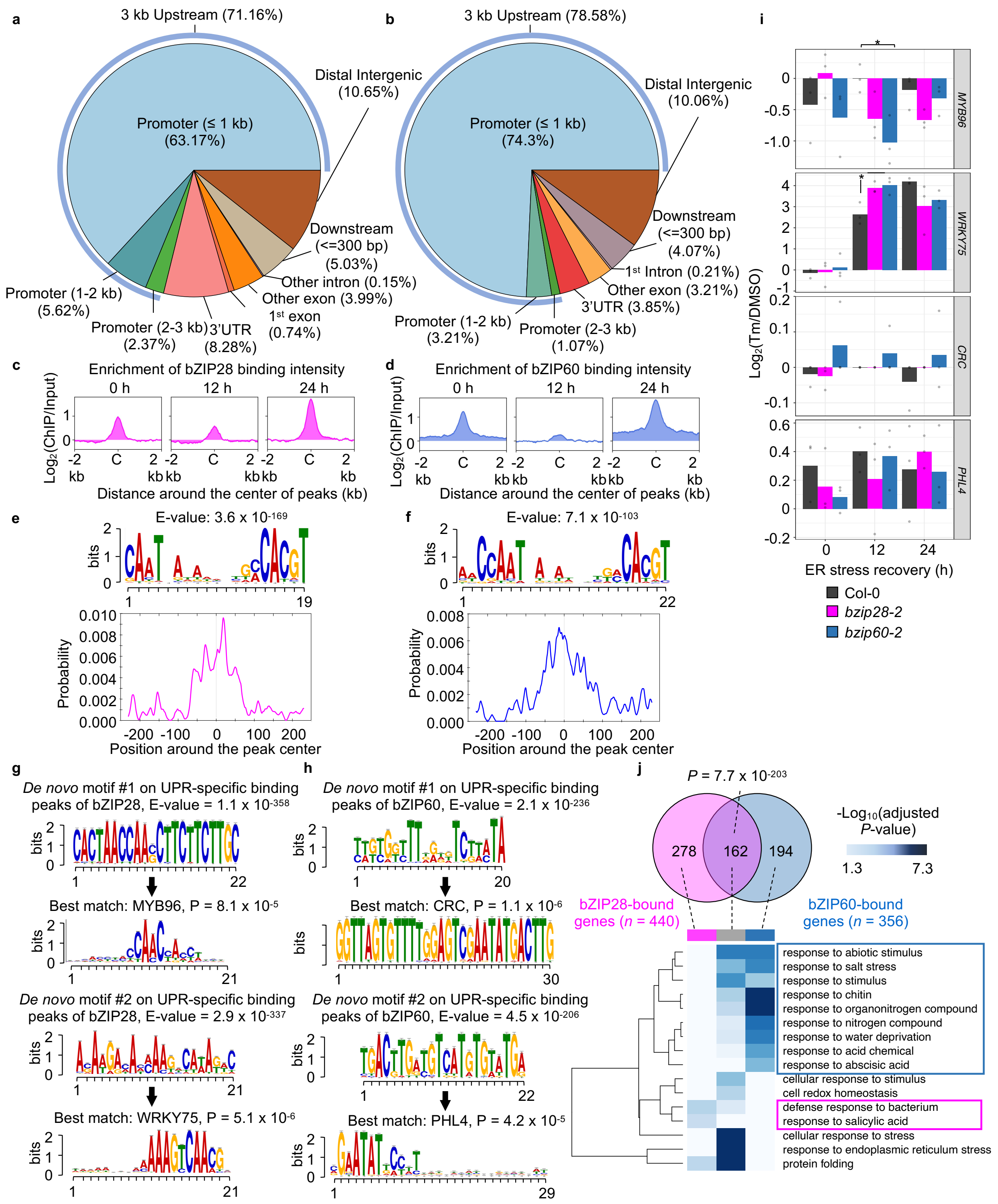

Supplementary Figure 4. Genome-wide DNA-binding profiles of bZIP28 and bZIP60 during ER stress recovery. a, b Distribution of bZIP28 and bZIP60 UPR-specific binding peaks relative to the gene structure. Pie charts depicting genomic features of binding peaks of bZIP28 (676 peaks) (a) and bZIP60 (467 peaks) (**b**). Most UPR-specific binding peaks (63.17%, bZIP28; 74.3%, bZIP60) were in the proximal gene promoters (i.e., within 1-kb of the transcription start site). **c, d** Metaplots depicting ChIP-seq signals of bZIP28 and bZIP60 at each time-point. Normalized enrichments were plotted around ± 2-kb from peak

centers. C: center of peaks. We observed similar temporal changes of bZIP28- and bZIP60-binding signals (input-normalized ChIP-seq signals), whereby an initial binding peak at 0 h was followed by a substantial decline at 12 h, and another peak at 24 h of ER stress recovery. **e, f** Enrichment of ERSE-I in UPR-specific binding peaks of bZIP28 (**e**) and bZIP60 (**f**). The CentriMo plots displayed the distribution of ERSE-I at the peak summits. Note that ERSE-I locates in ~10-bp right and left side of the summit of bZIP28 and bZIP60 UPR-specific binding peaks, respectively. **g, h** Top-ranked other TF motifs enriched in UPR-specific binding peaks of bZIP28 (**g**) and bZIP60 (**h**) are shown at the top along with E-value. The top matched motif in the Plant Cistromes Database[84] for each *de novo* motif is shown at the bottom along with *P*-value for statistical similarity. The analysis revealed other TF-binding motifs that were exclusively enriched in either bZIP28 or bZIP60 UPR-specific binding peaks. For example, TF binding motifs that were significantly similar to those of an R2R3-type MYB96 or WRKY75 were identified in bZIP28 UPR-specific binding peaks, but not in bZIP60 UPR-specific binding peaks. Conversely, TF binding motifs significantly similar to those of CRABS CLAW (CRC), a YABBY TF or Phosphate Starvation Response1-Like 4 (PHL4), a G2-like TF, were enriched only in bZIP60 UPR-specific binding peaks. **i** Expression changes represented by Log2(Tm/DMSO) of MYB96, WRKY75, CRC and PHL4 in each genotype at each time-point analyzed in the RNA-seq data. n = 3 (12 seedlings per replicate). *$P < 0.05$ (two-tailed Student's *t*-test). Interestingly, among them, the expression of MYB96 and WRKY75 were significantly altered in *bzip60-2* during ER stress recovery, suggesting a bZIP60-dependent transcriptional signal cascade affecting DNA-binding activities of bZIP28. **j** GO terms enriched in bZIP28-exclusively, shared and bZIP60-exclusively bound genes. The Venn diagram shows the number of bZIP28-exclusively, shared and bZIP60-exclusively bound genes. The total number of each set of genes is presented in parentheses. The adjusted *P*-value of the overlap between bZIP28- and bZIP60-bound genes was calculated using the hypergeometric test. GO terms associated with abiotic and biotic stress are marked by blue and magenta boxes, respectively. Among these genes, 162 genes were bound by both bZIP28 and bZIP60 (significant overlap validated by hypergeometric test, $P = 7.7 \times 10^{-203}$), including *BiP3* and *ERdj3B*, and showed a strong enrichment of GO terms associated with ER stress. In addition, 278 and 194 genes were exclusively bound by bZIP28 and bZIP60 and were enriched with distinct GO terms (e.g., biotic stress- vs. abiotic stress-related processes). A full list of GO terms is provided in Supplementary Data 5. The source data of **i, j** is provided in Supplementary Data 6.

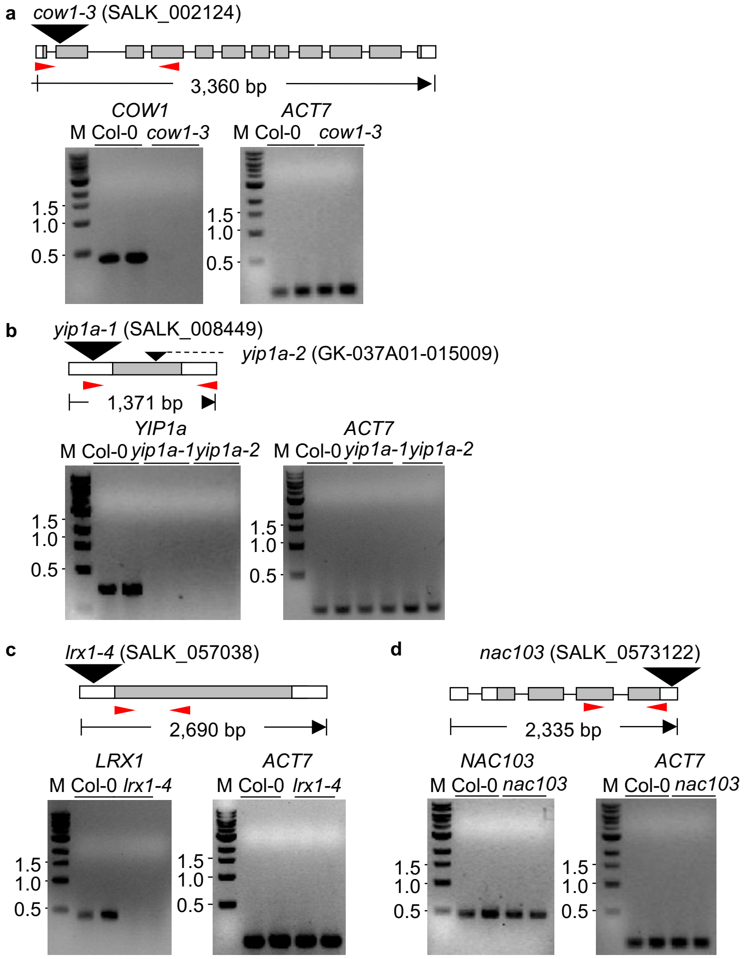

Supplementary Figure 5. Confirmation of gene knock-out in the loss-of-function mutants of predicted hub genes in the pink module. a-d Expression of the corresponding native gene in the mutant alleles of *COW1* (**a**), *Yip1a* (**b**), *LRX1* (**c**) and *NAC103* (**d**). A schematic representation of the T-DNA insertion is shown on the top. RT-PCR results on uncropped gels are shown at the bottom. *ACTIN7* (*ACT7*) serves as a loading control. PCR primer pairs are indicated by red arrowheads. M indicates the size marker. The DNA ladder size (kb) is indicated on the left of each uncropped gel.

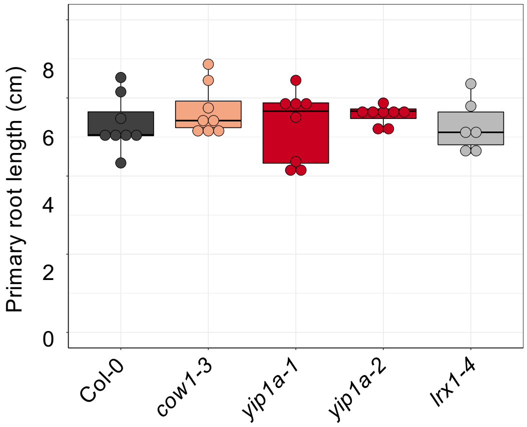

Supplementary Figure 6. Growth length of the primary root length of selected mutants for the pink module genes in normal conditions. Primary root growth in the loss-of-function mutants under normal conditions. *n* = 6-8. Seeds germinated and grew for 10 days under normal growth conditions. The source data is provided in Supplementary Data 6.

Supplementary Table 1. List of primers used in this study are shown below. Ori, orientation. F, forward. R, reverse.

Gene name	Gene ID	Sequence (5' to 3')	Ori	Description
SALK T-DNA LB1.3	-	ATTTTGCCGATTTCGGAAC	F	T-DNA specific primer
SAIL T-DNA Lb	-	GCCTTTTCAGAAATGGATAAATAGCCTT GCTTCC	F	T-DNA specific primer
WiscDsLox T-DNA	-	AACGTCCGCAATGTGTTATTAAGTTGTC	F	T-DNA specific primer
GABI-LB-8474	-	ATAATAACGCTGCGGACATCTACATTTT	F	T-DNA specific primer
bZIP28	AT3G10800	TTTATCATCATTTTGGTCGCC	F	Genotyping of bzip28-2, LP
bZIP28	AT3G10800	TATCCCCTAACAGGATACGGC	R	Genotyping of bzip28-2, RP
bZIP60	AT1G42990	GGAAGAAAAGTCCTCTCGGAG	F	Genotyping of bzip60-1, LP
bZIP60	AT1G42990	CACAGCATCATCGTCTCCTTC	R	Genotyping of bzip60-1, RP
bZIP60	AT1G42990	CAAAGATTGGCTCGTCTGAAC	F	Genotyping of bzip60-2, LP
bZIP60	AT1G42990	TGCTTGCGTATCTTGTGATATG	R	Genotyping of bzip60-2, RP
IRE1a	AT2G17520	CAAAATCTTCAGTGCTAGCGG	F	Genotyping of ire1a, LP
IRE1a	AT2G17520	TATCTCCGATCCATCGTTGAC	R	Genotyping of ire1a, RP
IRE1b	AT5G24360	CCTCTCGAACCCTTCAGGTAC	F	Genotyping of ire1b, LP
IRE1b	AT5G24360	GAAGGAAAACGGACATCCTTC	R	Genotyping of ire1b, RP
COW1	AT4G34580	TCTTTCCTCCATTGGATCATG	F	Genotyping of cow1-3, LP
COW1	AT4G34580	ATCATTTCTCATGCGATTTCG	R	Genotyping of cow1-3, RP
COW1	AT4G34580	ATGGCTGAGACCAAACCTG	F	RT-PCR
COW1	AT4G34580	CTCCATGATAGCCTTGAGGGT	R	RT-PCR
Yip1a	AT2G36300	ACCAAGCTCGTACACGTATGG	F	Genotyping of yip1a-1, LP
Yip1a	AT2G36300	TATCACGGTGGGAACGTAAAG	R	Genotyping of yip1a-1, RP
Yip1a	AT2G36300	GCAACATCAATTCCAATACTCTCA	F	Genotyping of yip1a-2, LP
Yip1a	AT2G36300	ATCTCTTTGCTGATTTGGTGAGAT	R	Genotyping of yip1a-2, RP
Yip1a	AT2G36300	TGAAACGTCACGAACGTGTG	F	RT-PCR
Yip1a	AT2G36300	TGGATGGATACCGAGCTCGT	R	RT-PCR
LRX1	AT1G12040	CTCCCTTTGCTTTGATGACAG	F	Genotyping lrx1-4, LP

LRX1	AT1G12040	ATTTGGTGTTGCTGATTCAGG	R	Genotyping of lrx1-4, RP
LRX1	AT1G12040	GCGACTTGGGCAGTGATATC	F	RT-PCR
LRX1	AT1G12040	CTCGTTGTAGCGGAGATCCA	R	RT-PCR
NAC103	AT5G64060	ACCAGTCTAGCTGCTAAGGCC	F	Genotyping of nac103, LP
NAC103	AT5G64060	GATGAAAGTCGCAAAGCAAAG	R	Genotyping of nac103, RP
NAC103	AT5G64060	GGTGGCGAGTTAAATTCGATG	F	RT-PCR
NAC103	AT5G64060	GCTCCACTACTGGACGGTTT	R	RT-PCR
YFP	-	AGCAGAAGAACGGCATCAA	F	qRT-PCR
YFP	-	GGGTGTTCTGCTGGTAGTG	R	qRT-PCR
bZIP28	AT3G10800	CGTCATCAGTCTCCAGCATTTC	F	qRT-PCR
bZIP28	AT3G10800	CTTGCCGTGGGTAGTGACATT	R	qRT-PCR
sbZIP60	AT1G42990	GGAGACGATGATGCTGTGGCT	F	qRT-PCR
sbZIP60	AT1G42990	CAGGGAACCCAACAGCAGACT	R	qRT-PCR
ubZIP60	AT1G42990	CAGGGATTCCAACAAGAGCACAG	R	qRT-PCR
BiP3	AT1G09080	CGAAACGTCTGATTGGAAGAA	F	qRT-PCR
BiP3	AT1G09080	GGCTTCCCATCTTTGTTCAC	R	qRT-PCR
ERdj3A	AT3G08970	GTGAAAGCGAAGAGCGTTGAT	F	qRT-PCR
ERdj3A	AT3G08970	TCACGCTGCTTTGCATCCT	R	qRT-PCR
ERdj3B	AT3G62600	GGAGGAGGCGGCATGAATAT	F	qRT-PCR
ERdj3B	AT3G62600	TCCATCGAACCTCCACCAAA	R	qRT-PCR
IRE1A	AT2G17520	AGACCCTGATTTACGTCCTAGC	F	qRT-PCR
IRE1A	AT2G17520	CCGACAAGTTCCTGAATTTCCG	R	qRT-PCR
PDIL1-1	AT1G21750	AAGTGGTCCTGCTTCTGTTGAA	F	qRT-PCR
PDIL1-1	AT1G21750	TTGAACAGCCTCACTGCAGGT	R	qRT-PCR
UBQ10	AT4G05320	GGCCTTGTATAATCCCTGATGAATAAG	F	qRT-PCR
UBQ10	AT4G05320	AAAGAGATAACAGGAACGGAAACATAGT	R	qRT-PCR
COW1	AT4G34580	AAGAGTTGACTGAGACCAAGAAG	F	qRT-PCR
COW1	AT4G34580	CAGCCTCTTGCTTTATGATCTCA	R	qRT-PCR
YIP1a	AT2G36300	TACGAGCTTGGTCGGATACT	F	qRT-PCR
YIP1a	AT2G36300	CAAAGCCGCAAGCACAAA	R	qRT-PCR
P58IPK	AT5G03160	TGACAGAGGAGAGGATCTTGA	F	qRT-PCR
P58IPK	AT5G03160	CCCTCGAAATGGAAAGTGTATTG	R	qRT-PCR
SEC31B	AT1G18830	AAACTCACTCAGCTCTGTCAAG	F	qRT-PCR
SEC31B	AT1G18830	AGAACTGCACTCATCCCATTC	R	qRT-PCR
BiP3	AT1G09080	CACGTGTCTGCTTGTGATTG	F	ChIP-qPCR
BiP3	AT1G09080	CGTATTTAGCCTCGGTAGAGTG	R	ChIP-qPCR
ERdj3B	AT3G62600	AGAACAGTCAGCGATTCGAC	F	ChIP-qPCR
ERdj3B	AT3G62600	TGCAATGCTTAACCAATCAGG	R	ChIP-qPCR
ERdj3B	AT3G62600	GGACCACTCGTGTGAGATG	F	ChIP-qPCR
ERdj3B	AT3G62600	GTCATATGTCAGGTCTCTTGATTCT	R	ChIP-qPCR
