# Supplementary material for: Advanced genomics identifies growth effectors for proteotoxic ER stress recovery in Arabidopsis thaliana
Source: Commun Biol. 2022 Jan 11;5:16. doi: 10.1038/s42003-021-02964-8 (PMC8752741; doi:10.1038/s42003-021-02964-8)
Supplement: Supplementary file 3 — Description of Additional Supplementary Files [file 42003_2021_2964_MOESM3_ESM.pdf]

Description of Additional Supplementary Files

File name: Supplementary Data 1
Description: The normalized read values (FPKM) for gene expression in all samples. Sample name consists of genotype, treatment, time-point and biological replicate with hyphen. WT, Col-0. 28, bzip28-2. 60, bzip60-2.

File name: Supplementary Data 2
Description: Log2-transformed fold-change (Tm/DMSO) of DEGs in all genotypes at all timepoints and coexpression module membership. Sample name consists of genotype, time-point and biological replicate with hyphen. WT, Col-0. 28, bzip28-2. 60, bzip60-2. A full list of DDEGs at each time-point. Pvalues (Student's t-test) calculated expression log2FC (Tm/DMSO) are shown next to Gene ID.

File name: Supplementary Data 3
Description: A full list of enriched GO terms for each of coexpression modules depicted in Fig. 3b.

File name: Supplementary Data 4
Description: UPR-specific binding peaks of bZIP28 and bZIP60 at 0, 12 and 24 h of ER stress recovery.

File name: Supplementary Data 5
Description: A full list of GO terms for target genes of UPR-bZIP TFs in Supplementary Figure 4j.

File name: Supplementary Data 6
Description: Growth length of the primary root length of selected mutants for the pink module genes in normal conditions.
