# Supplementary material for: Advanced genomics identifies growth effectors for proteotoxic ER stress recovery in Arabidopsis thaliana
Source: Commun Biol. 2022 Jan 11;5:16. doi: 10.1038/s42003-021-02964-8 (PMC8752741; doi:10.1038/s42003-021-02964-8)
Supplement: Supplementary file 10 — Reporting Summary [file 42003_2021_2964_MOESM10_ESM.pdf]

nature research

Corresponding author(s): Federica Brandizzi

Last updated by author(s): Nov 19, 2021

Reporting Summary

Nature Research wishes to improve the reproducibility of the work that we publish. This form provides structure for consistency and transparency in reporting. For further information on Nature Research policies, see our Editorial Policies and the Editorial Policy Checklist.

Statistics

For all statistical analyses, confirm that the following items are present in the figure legend, table legend, main text, or Methods section.

n/a	Confirmed	
☐	☒	The exact sample size (*n*) for each experimental group/condition, given as a discrete number and unit of measurement
☐	☒	A statement on whether measurements were taken from distinct samples or whether the same sample was measured repeatedly
☐	☒	The statistical test(s) used AND whether they are one- or two-sided<br>*Only common tests should be described solely by name; describe more complex techniques in the Methods section.*
☒	☐	A description of all covariates tested
☐	☒	A description of any assumptions or corrections, such as tests of normality and adjustment for multiple comparisons
☐	☒	A full description of the statistical parameters including central tendency (e.g. means) or other basic estimates (e.g. regression coefficient) AND variation (e.g. standard deviation) or associated estimates of uncertainty (e.g. confidence intervals)
☐	☒	For null hypothesis testing, the test statistic (e.g. *F*, *t*, *r*) with confidence intervals, effect sizes, degrees of freedom and *P* value noted<br>*Give P values as exact values whenever suitable.*
☒	☐	For Bayesian analysis, information on the choice of priors and Markov chain Monte Carlo settings
☐	☒	For hierarchical and complex designs, identification of the appropriate level for tests and full reporting of outcomes
☒	☐	Estimates of effect sizes (e.g. Cohen's *d*, Pearson's *r*), indicating how they were calculated

Our web collection on statistics for biologists contains articles on many of the points above.

Software and code

Policy information about availability of computer code

Data collection	No special software was used for data collection.
Data analysis	RNA-seq: The quality of raw reads was evaluated using FastQC (version 0.11.5). Reads were cleaned for quality and adapters with Cutadapt (version 1.8.1) using a minimum base quality of 20 retaining reads with a minimum length of 30 nucleotides after trimming. Quality-filtered reads were aligned to the Col-0 reference genome (TAIR10) using Bowtie (version 2.2.4) and TopHat (version 2.0.14) with a 10 bp minimum intron length and 15,000 bp maximum intron length. Fragments per kilobase exon model per million mapped reads (FPKM) were calculated using TAIR10 gene model annotation with Cufflinks (version 1.3.0). Per-gene read counts were measured using HTSeq (version 0.6.1p1) in the union mode with a minimum mapping quality of 20 with stranded=reverse counting. Differential gene expression analysis was performed in each sample relative to the mock control using DESeq2 (version 1.16.1) within R (version 3.4.0). Genes of which the total count is < 100 were not included in the analysis. DEGs were obtained based on adjusted P-value < 0.01 and absolute Log2FC > 1. Among DEGs identified in one or more genotypes at one or more time-points (n = 6670), DDEGs were obtained based the pairwise comparison of each DEG between WT and bzip28, WT and bzip60 or bzip28 and bzip60 with two-tailed Student t-test P < 0.01. For visualization purpose, tdf files of each replicate file were generated using igv tools (version 2.3.26) with the command "count" and loaded to Integrative Genome Browser (version 2.5.0). Gene ontology (GO) enrichment analysis was performed using agriGO (version 2.0) (http://systemsbiology.cau.edu.cn/agriGOv2/)75 with a false-discovery rate adjusted P < 0.05 (hypergeometric test) as a cutoff. Biological process GO categories were analyzed and the heatmap of GO analysis was produced using R package ggplots. To validate the RNA-seq profiling, we performed quantitative RT-PCR (qRT-PCR) for 11 genes including seven known UPR biomarker genes and four downstream genes identified or will be identified below (Supplementary Fig. 3b). We generated a total of 93 data points (Log2[Tm/DMSO]) across the time-points and genotypes, which significantly correlated with those measured by the RNA-seq analysis (PCC = 0.947, P < 2.2 x 10-16).<br><br>ChIP-seq: The quality of raw ChIP-seq reads was evaluated using FastQC (version 0.11.5). Reads were cleaned for quality and adapters with Cutadapt (version 1.8.1)68 using a minimum base quality of 20 retaining reads with a minimum length of 30 nucleotides after trimming. Quality-filtered reads were aligned to the Col-0 reference genome (TAIR10) using Bowtie (version 1.1.2) with parameters "-n 2 -m 3 -k 1 --

threads 7 --best --chunkmbs 256 -q". Duplicated reads were removed using Samtools (version 1.8)78. Peak calling was performed using MACS2 (version 2.1.2) in individual samples with input samples pooled for DMSO-only and Tm treated samples and with a relaxed threshold of P-value (--pvalue=1e-2), as recommended by the IDR pipeline (https://sites.google.com/site/anshulkundaje/projects/idr). Peaks across replicates with an IDR < 0.05 were retained for further analysis. To obtain binding peaks with high-confidence, we applied two parameters to IDR-filtered peaks; (1) a peak called in Tm samples that was overlapped with a peak in the corresponding DMSO-only samples by > 30% was eliminated, (2) Among the peaks that were overlapped with peaks in DMSO-only sample, if its p-value float (8th column in the IDR output file) in the Tm treated sample was higher than the corresponding peak in the DMSO-only treated sample by greater than 3-fold, the peak was retained and named as a UPR-specific binding peak. UPR-specific binding peaks obtained at each time-point were merged into a single list for further analysis and were annotated using the ChIPseeker and the GenomicFeatures R Package. For visualization purpose, bigwig files (using pooled data across biological replicates) were generated by deepTools suite (https://deeptools.readthedocs.io/en/develop/) with the command "bamCoverage"; read coverage was normalized as RPKM (Reads Per Kilobase per Million reads). ChIP-seq tracks were visualized in Integrative Genome Browser (version 2.5.0). ChIP-seq metaplot was generated from the merged single file by deepTools suite with the commands "bamCompare" and "plotProfile". UPR-specific binding peaks were mapped to the vicinity of a coding sequence (<1 kb), generating a total of 440 bZIP28- and 356 bZIP60-bound genes, respectively. Gene ontology (GO) enrichment analysis was performed as described in RNA-seq analysis.

All other relevant data supporting findings of this paper are available upon request.

For manuscripts utilizing custom algorithms or software that are central to the research but not yet described in published literature, software must be made available to editors and reviewers. We strongly encourage code deposition in a community repository (e.g. GitHub). See the Nature Research guidelines for submitting code & software for further information.

Data

Policy information about availability of data

All manuscripts must include a data availability statement. This statement should provide the following information, where applicable:

- Accession codes, unique identifiers, or web links for publicly available datasets
- A list of figures that have associated raw data
- A description of any restrictions on data availability

RNA-seq and ChIP-seq sequencing data is available at the National Center for Biotechnology Information Gene Expression Omnibus (NCBI GEO), with Accession number GSE146723. Processed data supporting the findings of this paper also are available within the paper and its Supplementary Information files.

Field-specific reporting

Please select the one below that is the best fit for your research. If you are not sure, read the appropriate sections before making your selection.

☒ Life sciences ☐ Behavioural & social sciences ☐ Ecological, evolutionary & environmental sciences

For a reference copy of the document with all sections, see nature.com/documents/nr-reporting-summary-flat.pdf

Life sciences study design

All studies must disclose on these points even when the disclosure is negative.

Sample size	For RNA-seq analyses, a total of 54 samples, which consist of 3 genotypes under 2 treatments at 3 time-points in 3 biological replicates, were processed. For ChIP-seq analyses, a total of 48 samples, which consist of 2 proteins under 2 treatment at 3 time-points in 2 biological replicates of each sample type (ChIP or input), were processed. For LC/MS/MS analyses, a total of 38 samples, which consists of 30 samples tested and 8 analytic control (standard curve) samples, were processed.
Data exclusions	No data were excluded from analysis.
Replication	For each RNA-seq experiments, we performed three biological replicates. For the ChIP-seq, two biological replicates were performed.
Randomization	Plants were grown in a randomized manner and were rotated every other days in the plant growth incubator. Biological replicates, each of which consisted of randomly pooled plants, were randomly selected.
Blinding	Blinding was not used because our analyses were not vulnerable to a observer bias. Randomized methods were applied to the sample collection. All data including mock controls (i.e., the corresponding DMSO-only samples) were processed in parallel.

Reporting for specific materials, systems and methods

We require information from authors about some types of materials, experimental systems and methods used in many studies. Here, indicate whether each material, system or method listed is relevant to your study. If you are not sure if a list item applies to your research, read the appropriate section before selecting a response.

Materials & experimental systems

n/a	Involved in the study
☐	☒ Antibodies
☐	☐ Eukaryotic cell lines
☐	☐ Palaeontology and archaeology
☐	☐ Animals and other organisms
☐	☐ Human research participants
☐	☐ Clinical data
☐	☐ Dual use research of concern

Methods

n/a	Involved in the study
☐	☒ ChIP-seq
☐	☐ Flow cytometry
☐	☐ MRI-based neuroimaging

Antibodies

Antibodies used: Anti-GFP (Abcam, cat# ab290) was used for ChIP.

Validation: The specificity and suitability of the antibodies for ChIP were tested by the supplier. They were verified in earlier publications for ChIP analyses, as indicated in the manuscript.

Eukaryotic cell lines

Policy information about cell lines

Cell line source(s): *State the source of each cell line used.*

Authentication: *Describe the authentication procedures for each cell line used OR declare that none of the cell lines used were authenticated.*

Mycoplasma contamination: *Confirm that all cell lines tested negative for mycoplasma contamination OR describe the results of the testing for mycoplasma contamination OR declare that the cell lines were not tested for mycoplasma contamination.*

Commonly misidentified lines (See ICLAC register): *Name any commonly misidentified cell lines used in the study and provide a rationale for their use.*

Palaeontology and Archaeology

Specimen provenance: *Provide provenance information for specimens and describe permits that were obtained for the work (including the name of the issuing authority, the date of issue, and any identifying information).*

Specimen deposition: *Indicate where the specimens have been deposited to permit free access by other researchers.*

Dating methods: *If new dates are provided, describe how they were obtained (e.g. collection, storage, sample pretreatment and measurement), where they were obtained (i.e. lab name), the calibration program and the protocol for quality assurance OR state that no new dates are provided.*

☐ Tick this box to confirm that the raw and calibrated dates are available in the paper or in Supplementary Information.

Ethics oversight: *Identify the organization(s) that approved or provided guidance on the study protocol, OR state that no ethical approval or guidance was required and explain why not.*

Note that full information on the approval of the study protocol must also be provided in the manuscript.

Animals and other organisms

Policy information about studies involving animals; ARRIVE guidelines recommended for reporting animal research

Laboratory animals: *For laboratory animals, report species, strain, sex and age OR state that the study did not involve laboratory animals.*

Wild animals: *Provide details on animals observed in or captured in the field; report species, sex and age where possible. Describe how animals were caught and transported and what happened to captive animals after the study (if killed, explain why and describe method; if released, say where and when) OR state that the study did not involve wild animals.*

Field-collected samples: *For laboratory work with field-collected samples, describe all relevant parameters such as housing, maintenance, temperature, photoperiod and end-of-experiment protocol OR state that the study did not involve samples collected from the field.*

Ethics oversight: *Identify the organization(s) that approved or provided guidance on the study protocol, OR state that no ethical approval or guidance was required and explain why not.*

Note that full information on the approval of the study protocol must also be provided in the manuscript.

Human research participants

Policy information about studies involving human research participants

Population characteristics	*Describe the covariate-relevant population characteristics of the human research participants (e.g. age, gender, genotypic information, past and current diagnosis and treatment categories). If you filled out the behavioural & social sciences study design questions and have nothing to add here, write "See above."*
Recruitment	*Describe how participants were recruited. Outline any potential self-selection bias or other biases that may be present and how these are likely to impact results.*
Ethics oversight	*Identify the organization(s) that approved the study protocol.*

Note that full information on the approval of the study protocol must also be provided in the manuscript.

Clinical data

Policy information about clinical studies

All manuscripts should comply with the ICMJE guidelines for publication of clinical research and a completed CONSORT checklist must be included with all submissions.

Clinical trial registration	*Provide the trial registration number from ClinicalTrials.gov or an equivalent agency.*
Study protocol	*Note where the full trial protocol can be accessed OR if not available, explain why.*
Data collection	*Describe the settings and locales of data collection, noting the time periods of recruitment and data collection.*
Outcomes	*Describe how you pre-defined primary and secondary outcome measures and how you assessed these measures.*

Dual use research of concern

Policy information about dual use research of concern

Hazards

Could the accidental, deliberate or reckless misuse of agents or technologies generated in the work, or the application of information presented in the manuscript, pose a threat to:

No	Yes	
☐	☐	Public health
☐	☐	National security
☐	☐	Crops and/or livestock
☐	☐	Ecosystems
☐	☐	Any other significant area

Experiments of concern

Does the work involve any of these experiments of concern:

No	Yes	
☐	☐	Demonstrate how to render a vaccine ineffective
☐	☐	Confer resistance to therapeutically useful antibiotics or antiviral agents
☐	☐	Enhance the virulence of a pathogen or render a nonpathogen virulent
☐	☐	Increase transmissibility of a pathogen
☐	☐	Alter the host range of a pathogen
☐	☐	Enable evasion of diagnostic/detection modalities
☐	☐	Enable the weaponization of a biological agent or toxin
☐	☐	Any other potentially harmful combination of experiments and agents

ChIP-seq

Data deposition

☒ Confirm that both raw and final processed data have been deposited in a public database such as GEO.

☒ Confirm that you have deposited or provided access to graph files (e.g. BED files) for the called peaks.

Data access links
May remain private before publication.

https://www.ncbi.nlm.nih.gov/geo/query/acc.cgi?acc=GSE146723

Files in database submission

28-0-D1_chip.fastq.gz
28-0-D1_input.fastq.gz
28-0-D2_chip.fastq.gz
28-0-D2_input.fastq.gz
28-0-T1_chip.fastq.gz
28-0-T1_input.fastq.gz
28-0-T2_chip.fastq.gz
28-0-T2_input.fastq.gz
28-12-D1_chip.fastq.gz
28-12-D1_input.fastq.gz
28-12-D2_chip.fastq.gz
28-12-D2_input.fastq.gz
28-12-T1_chip.fastq.gz
28-12-T1_input.fastq.gz
28-12-T2_chip.fastq.gz
28-12-T2_input.fastq.gz
28-24-D1_chip.fastq.gz
28-24-D1_input.fastq.gz
28-24-D2_chip.fastq.gz
28-24-D2_input.fastq.gz
28-24-T1_chip.fastq.gz
28-24-T1_input.fastq.gz
28-24-T2_chip.fastq.gz
28-24-T2_input.fastq.gz
60-0-D1_chip.fastq.gz
60-0-D1_input.fastq.gz
60-0-D2_chip.fastq.gz
60-0-D2_input.fastq.gz
60-0-T1_chip.fastq.gz
60-0-T1_input.fastq.gz
60-0-T2_chip.fastq.gz
60-0-T2_input.fastq.gz
60-12-D1_chip.fastq.gz
60-12-D1_input.fastq.gz
60-12-D2_chip.fastq.gz
60-12-D2_input.fastq.gz
60-12-T1_chip.fastq.gz
60-12-T1_input.fastq.gz
60-12-T2_chip.fastq.gz
60-12-T2_input.fastq.gz
60-24-D1_chip.fastq.gz
60-24-D1_input.fastq.gz
60-24-D2_chip.fastq.gz
60-24-D2_input.fastq.gz
60-24-T1_chip.fastq.gz
60-24-T1_input.fastq.gz
60-24-T2_chip.fastq.gz
60-24-T2_input.fastq.gz
ChIPseq_bZIP28_peaks_0h.txt
ChIPseq_bZIP28_peaks_12h.txt
ChIPseq_bZIP28_peaks_24h.txt
ChIPseq_bZIP60_peaks_0h.txt
ChIPseq_bZIP60_peaks_12h.txt
ChIPseq_bZIP60_peaks_24h.txt

Genome browser session
(e.g. UCSC)

Provide a link to an anonymized genome browser session for "Initial submission" and "Revised version" documents only, to enable peer review. Write "no longer applicable" for "Final submission" documents.

Methodology

Replicates

Two biological replicates for each genotype in each treatment at each time-point

Sequencing depth

Sample name/total reads/uniquely mapped reads/uniquely mapped reads with no duplicates
28-0-D1_chip /44148143 /16846548 /2553470
28-0-D1_input /42264802 /28969038 /7046455
28-0-D2_chip /23603788 /8284776 /2042850
28-0-D2_input /26543124 /15786175 /2641338
28-0-T1_chip /55700654 /18667074 /4276563
28-0-T1_input /17583009 /10523445 /2242972
28-0-T2_chip /26712703 /9923667 /1983878
28-0-T2_input /28904792 /17740126 /3640721

28-12-D1_chip /19836986 /7741039 /1933067
28-12-D1_input /26409009 /18659503 /9455629
28-12-D2_chip /55749477 /19451899 /4832598
28-12-D2_input /32348659 /22306045 /8035143
28-12-T1_chip /26832625 /10166532 /2701406
28-12-T1_input /58332068 /40437871 /16753774
28-12-T2_chip /12018778 /5304635 /1982326
28-12-T2_input /43737805 /29588249 /5323839
28-24-D1_chip /43034486 /10998431 /2816169
28-24-D1_input /38827694 /25425635 /6255733
28-24-D2_chip /25864332 /12928045 /4236575
28-24-D2_input /10423223 /6249374 /859069
28-24-T1_chip /51930559 /12224128 /3404316
28-24-T1_input /30417747 /19926967 /5197252
28-24-T2_chip /51295521 /8974432 /1763921
28-24-T2_input /27904076 /18726034 /4308180
60-0-D1_chip /40520612 /13598924 /4842123
60-0-D1_input /51635002 /32591497 /23404650
60-0-D2_chip /21810947 /11686069 /3312274
60-0-D2_input /35533036 /24196861 /17591831
60-0-T1_chip /29650560 /13667454 /5055080
60-0-T1_input /32927781 /20968476 /14700534
60-0-T2_chip /51602607 /26347792 /8778267
60-0-T2_input /57182659 /35727012 /24846334
60-12-D1_chip /24347852 /15454707 /5454818
60-12-D1_input /43927645 /30587086 /19791819
60-12-D2_chip /19199539 /11882404 /3994269
60-12-D2_input /32626063 /22801610 /14778342
60-12-T1_chip /21490814 /13172338 /4809842
60-12-T1_input /44057132 /26649197 /3738444
60-12-T2_chip /19125615 /11438210 /4783319
60-12-T2_input /44836866 /32426468 /9004895
60-24-D1_chip /24763274 /11493912 /2160511
60-24-D1_input /51204633 /35256683 /5048838
60-24-D2_chip /36091332 /11177368 /1739023
60-24-D2_input /31412395 /21057239 /3454088
60-24-T1_chip /35031391 /17352300 /3143415
60-24-T1_input /13921027 /9468498 /1246391
60-24-T2_chip /34933182 /15795200 /2901446
60-24-T2_input /12604088 /5359666 /1359975

Antibodies

Anti-GFP (Abcam, cat# ab290) was used for ChIP.

Peak calling parameters

Peak calling was performed using MACS2 (version 2.1.2) in individual samples with input samples pooled for DMSO-only and Tm treated samples and with a relaxed threshold of P-value (--pvalue=1e-2), as recommended by the IDR pipeline (https://sites.google.com/site/anshulkundaje/projects/idr).

Data quality

Peaks across replicates with an IDR < 0.05 were retained for further analysis. To obtain binding peaks with high-confidence, we applied two parameters to IDR-filtered peaks; (1) a peak called in Tm samples that was overlapped with a peak in the corresponding DMSO-only samples by > 30% was eliminated, (2) Among the peaks that were overlapped with peaks in DMSO-only sample, if its p-value float (8th column in the IDR output file) in the Tm treated sample was higher than the corresponding peak in the DMSO-only treated sample by greater than 3-fold, the peak was retained and named as a UPR-specific binding peak.

Software

UPR-specific binding peaks obtained at each time-point were merged into a single list for further analysis and were annotated using the ChIPseeker and the GenomicFeatures R Package.

Flow Cytometry

Plots

Confirm that:

☐ The axis labels state the marker and fluorochrome used (e.g. CD4-FITC).

☐ The axis scales are clearly visible. Include numbers along axes only for bottom left plot of group (a 'group' is an analysis of identical markers).

☐ All plots are contour plots with outliers or pseudocolor plots.

☐ A numerical value for number of cells or percentage (with statistics) is provided.

Methodology

Sample preparation

Describe the sample preparation, detailing the biological source of the cells and any tissue processing steps used.

Instrument	*Identify the instrument used for data collection, specifying make and model number.*
Software	*Describe the software used to collect and analyze the flow cytometry data. For custom code that has been deposited into a community repository, provide accession details.*
Cell population abundance	*Describe the abundance of the relevant cell populations within post-sort fractions, providing details on the purity of the samples and how it was determined.*
Gating strategy	*Describe the gating strategy used for all relevant experiments, specifying the preliminary FSC/SSC gates of the starting cell population, indicating where boundaries between "positive" and "negative" staining cell populations are defined.*

☐ Tick this box to confirm that a figure exemplifying the gating strategy is provided in the Supplementary Information.

Magnetic resonance imaging

Experimental design

Design type	*Indicate task or resting state; event-related or block design.*
Design specifications	*Specify the number of blocks, trials or experimental units per session and/or subject, and specify the length of each trial or block (if trials are blocked) and interval between trials.*
Behavioral performance measures	*State number and/or type of variables recorded (e.g. correct button press, response time) and what statistics were used to establish that the subjects were performing the task as expected (e.g. mean, range, and/or standard deviation across subjects).*

Acquisition

Imaging type(s)	*Specify: functional, structural, diffusion, perfusion.*
Field strength	*Specify in Tesla*
Sequence & imaging parameters	*Specify the pulse sequence type (gradient echo, spin echo, etc.), imaging type (EPI, spiral, etc.), field of view, matrix size, slice thickness, orientation and TE/TR/flip angle.*
Area of acquisition	*State whether a whole brain scan was used OR define the area of acquisition, describing how the region was determined.*

Diffusion MRI ☐ Used ☐ Not used

Preprocessing

Preprocessing software	*Provide detail on software version and revision number and on specific parameters (model/functions, brain extraction, segmentation, smoothing kernel size, etc.).*
Normalization	*If data were normalized/standardized, describe the approach(es): specify linear or non-linear and define image types used for transformation OR indicate that data were not normalized and explain rationale for lack of normalization.*
Normalization template	*Describe the template used for normalization/transformation, specifying subject space or group standardized space (e.g. original Talairach, MNI305, ICBM152) OR indicate that the data were not normalized.*
Noise and artifact removal	*Describe your procedure(s) for artifact and structured noise removal, specifying motion parameters, tissue signals and physiological signals (heart rate, respiration).*
Volume censoring	*Define your software and/or method and criteria for volume censoring, and state the extent of such censoring.*

Statistical modeling & inference

Model type and settings	*Specify type (mass univariate, multivariate, RSA, predictive, etc.) and describe essential details of the model at the first and second levels (e.g. fixed, random or mixed effects; drift or auto-correlation).*
Effect(s) tested	*Define precise effect in terms of the task or stimulus conditions instead of psychological concepts and indicate whether ANOVA or factorial designs were used.*

Specify type of analysis: ☐ Whole brain ☐ ROI-based ☐ Both

Statistic type for inference (See Eklund et al. 2016)	*Specify voxel-wise or cluster-wise and report all relevant parameters for cluster-wise methods.*
Correction	*Describe the type of correction and how it is obtained for multiple comparisons (e.g. FWE, FDR, permutation or Monte Carlo).*

Models & analysis

n/a	Involved in the study
☐	☐ Functional and/or effective connectivity
☐	☐ Graph analysis
☐	☐ Multivariate modeling or predictive analysis

Functional and/or effective connectivity	*Report the measures of dependence used and the model details (e.g. Pearson correlation, partial correlation, mutual information).*
Graph analysis	*Report the dependent variable and connectivity measure, specifying weighted graph or binarized graph, subject- or group-level, and the global and/or node summaries used (e.g. clustering coefficient, efficiency, etc.).*
Multivariate modeling and predictive analysis	*Specify independent variables, features extraction and dimension reduction, model, training and evaluation metrics.*
